# Correlation Analysis between Dietary Intake of Tyrosols and Their Food Sources and Urinary Excretion of Tyrosol and Hydroxytyrosol in a European Population

**DOI:** 10.3390/antiox12030715

**Published:** 2023-03-14

**Authors:** Enrique Almanza-Aguilera, Estefanía Davila-Cordova, Daniel Guiñón-Fort, Marta Farràs, Giovanna Masala, Maria Santucci de Magistris, Ivan Baldassari, Rosario Tumino, Lisa Padroni, Verena A Katzke, Matthias B. Schulze, Augustin Scalbert, Raul Zamora-Ros

**Affiliations:** 1 Unit of Nutrition and Cancer, Cancer Epidemiology Research Program, Catalan Institute of Oncology (ICO), Bellvitge Biomedical Research Institute (IDIBELL), 08908 L’Hospitalet de Llobregat, Spain; 2 Cancer Risk Factors and Life-Style Epidemiology Unit, Institute for Cancer Research, Prevention and Clinical Network (ISPRO), 50139 Florence, Italy; 3 Dipartimento di Medicina Clinica e Chirurgia, Federico II, 80131 Naples, Italy; 4 Department of Epidemiology and Data Science, Fondazione IRCCS Istituto Nazionale dei Tumori di Milano, 20133 Milan, Italy; 5 Cancer Registry and Histopathology Department, Provincial Health Authority (ASP 7), 97100 Ragusa, Italy; 6 Unit of Cancer Epidemiology, Città della Salute e della Scienza University, Hospital and Center for Cancer Prevention (CPO), Via Santena 7, 10126 Turin, Italy; 7 Division of Cancer Epidemiology, German Cancer Research Center (DKFZ), 69120 Heidelberg, Germany; 8 Department of Molecular Epidemiology, German Institute of Human Nutrition Potsdam-Rehbruecke, 14558 Nuthetal, Germany; 9 Institute of Nutritional Science, University of Potsdam, 14558 Nuthetal, Germany; 10 Nutrition and Metabolism Branch, International Agency for Research on Cancer (IARC-WHO), 69372 Lyon, France

**Keywords:** tyrosol, hydroxytyrosol, urine, olive oil, wine, biomarkers, EPIC

## Abstract

This study analyzed the correlations between the acute and habitual intake of dietary tyrosols, their main food sources, and 24 h urine excretions of tyrosol (Tyr) and hydroxytyrosol (OHTyr) in participants from the European Prospective Investigation into Cancer and Nutrition study (EPIC). Participants (n = 419) were healthy men and women aged from 34 to 73 years from 8 EPIC centers belonging to France, Italy, and Germany. Acute and habitual dietary data were collected using a standardized 24 h dietary recall software and validated country-specific dietary questionnaires, respectively. The intake of 13 dietary tyrosols was estimated using the Phenol-Explorer database. Excretions of Tyr and OHTyr in a single 24 h urine sample were analyzed using tandem mass spectrometry. Urinary excretions of Tyr, OHTyr, and their sum (Tyr + OHTyr) correlated more strongly with their corresponding acute (rho_partial_~0.63) rather than habitual intakes (rho_partial_~0.47). In addition, individual and combined urinary excretions of Tyr and OHTyr were weakly to moderately correlated with the acute and habitual intake of other individual tyrosol precursors (rho_partial_ = 0.10–0.44) and especially with major food sources, such as wine (rho_partial_ = 0.41–0.58), olive oil (rho_partial_ = 0.25–0.44), and beer (rho_partial_ = 0.14–0.23). Urinary Tyr + OHTyr excretions were similarly correlated with the acute intake of total tyrosols but differently correlated with food sources among countries. Based on these results, we conclude that 24 h urinary excretions of Tyr + OHTyr could be proposed as biomarkers of total tyrosol intake, preferably for acute intakes.

## 1. Introduction

Tyrosol (Tyr) and hydroxytyrosol (OHTyr) are the most representative (poly)phenols of olives and olive oil, where they can be found as either free (aglycones) or conjugated forms, such as secoiridoid glycosides (e.g., oleuropein and ligstroside) and secoiridoid aglycones (e.g., oleacein and oleocanthal) [1]. Together, Tyr, OHTyr, and their precursor compounds are referred to as tyrosols. Tyr and OHTyr can also be found in wine and beer, but their excretions in these fermented beverages are usually lower than in olives and olive oil, especially OHTyr [2,3]. Tyrosols are less consumed than other (poly)phenol subclasses; the mean tyrosol intake in European adults is approximately 12 mg/d, Tyr, oleuropein-aglycone di-aldehyde (3,4-DHPEA-EDA) and oleuropein aglycone being their main contributors [4].

Despite being well absorbed, the dietary forms of tyrosols are poorly found in either urine or blood samples due to being subjected to extensive phase I/II and microbial metabolism in the human body. To date, more than 10 metabolites of Tyr and OHTyr have been found in several pharmacokinetic animal and human studies, including O-methylated forms, aldehydes, and acids formed via oxidation of the aliphatic alcohol, sulfates, glucuronides, acetylated, sulfated, and an N-acetylcysteine derivative [5,6]. The results from those studies suggested that the absorption of Tyr and OHTyr also depends on the composition of the food matrix [2,7,8]. Indeed, recent research demonstrated that, after wine and beer consumption, Tyr is absorbed and partially bio-transformed into OHTyr [2,9]. Likewise, small quantities of Tyr and OHTyr can also be endogenously synthesized in the human body as byproducts of dopamine and tyramine metabolisms, respectively [5].

In addition, tyrosols can exert important biological effects on human health, having antioxidant, anti-inflammatory, antiatherogenic, cardioprotective, anticancer, neuroprotective, antidiabetic, and antiobesity effects, among others [8,10]. In fact, in vitro studies have demonstrated that OHTyr possesses a higher antioxidant capacity than Tyr due to the lack of a catechol group (-OH) in the latter [11]. However, evidence from epidemiological studies is lacking, probably because of the difficulty in accurately estimating tyrosol exposures.

Currently, the estimation of tyrosol intake is based on either the combined use of self-reported questionnaires and (poly)phenol food composition databases [4,12] or the quantification of Tyr and OHTyr in biological fluids [13,14]. To date, however, these methods have hardly been used together to evaluate the potential use of systemic (poly)phenol excretions as biomarkers of their dietary intake, especially in large populations [15]. In this regard, previous small studies have shown that 24 h urinary excretions of Tyr and OHTyr are strongly correlated (r_Pearson_ > 0.70) with the controlled acute intakes of these compounds [13,16]. To date, however, there are no previously published works on the correlations between 24 h excretions of Tyr + OHTyr and the acute and chronic dietary intakes of these and other tyrosols.

In the current study, we aimed to assess the correlation between acute and habitual dietary tyrosol intake, their main dietary sources, and 24 h urine excretions of Tyr and OHTyr in individuals from a European population. Although such correlations would be somehow expected from a biological point of view, with this research, we propose to explore not only their strength and statistical significance but also the potential differences between acute and habitual intake periods.

## 2. Materials and Methods

### 2.1. Study Population

The European Prospective Investigation into Cancer and Nutrition (EPIC) study is an ongoing large prospective multi-center cohort [17,18]. The participants of the EPIC study include healthy adult men and women (n = 521,000) recruited mostly from the general population between 1992 and 2000 across 10 European countries. The ethical approval for the EPIC study was obtained from the ethical review boards of the International Agency for Research on Cancer (IARC, Lyon, France) and all the participating EPIC centers. All participants provided written informed consent.

### 2.2. Samples and Analytical Method

The current EPIC substudy includes a convenience subsample (i.e., participants with a single 24 h dietary recall (24-HDR) and 24 h urine samples collected the same day), composed of 419 women and men, aged between 34 and 73 years, and recruited from 8 EPIC centers (i.e., Paris, Florence, Varese, Ragusa, Turin, Naples, Heidelberg, and Potsdam). It should be noted that the participants from Paris and Naples centers were all women. Participants’ data from Greece were not available for the current study and were therefore excluded compared to previous EPIC studies [19]. Collection, storage, and analysis of urine samples were carried out as previously described [20]. Briefly, urine samples were collected over a 24 h period, submitted to the EPIC center the same day as the 24-HDR interview, and stored at −20 °C using boric acid as a preservative. The completeness of 24 h urine sampling was verified using p-aminobenzoic acid; samples with a p-aminobenzoic acid recovery between 70% and 110% were retained for further analysis. The analysis of Tyr and OHTyr in urine samples was conducted in an ultra-performance liquid chromatography–tandem mass spectrometry system (UPLC-MS/MS) with prior enzymatic hydrolysis. All phenolic groups in Tyr and OHTyr were quantitatively marked using a differential isotope-labeling method. The limits of quantification (LOQ) for Tyr and OHTyr were 0.02 and 0.23 μM, respectively. The intra-batch and inter-batch coefficients of variability (CV) were 4.1 and 7.5 for Tyr, and 4.9 and 7.9 for OHTyr. The urinary excretion of both tyrosols was expressed as μmol/24 h; total urinary tyrosols were calculated as the sum of Tyr and OHTyr (Tyr + OHTyr).

### 2.3. Dietary and Lifestyle Information

Data on the acute and habitual intake of foods were collected using a single 24-HDR [21], and a quantitative or semi-quantitative dietary questionnaire (DQ) [17,18], respectively. The 24-HDR was administered in a face-to-face interview using the standardized software EPIC-Soft (renamed GloboDiet) [21]. The DQs were developed and validated in each EPIC center/country [17]. The average time interval between the collection of the 24-HDR and the DQ varied between one and three years [22]. The dietary intakes of the following 13 tyrosols were estimated using the Phenol-Explorer database [23]: Tyr, OHTyr, Tyr acetate (p-HPEA-AC), OHTyr acetate (3,4-DHPEA-AC), oleuropein, oleuropein aglycone, oleuropein-aglycone mono-aldehyde (3,4-DHPEA-EA), oleuropein-aglycone di-aldehyde (3,4-DHPEA-EDA), ligstroside, ligstroside aglycone, ligstroside-aglycone mono-aldehyde (p-HPEA-EA), ligstroside-aglycone di-aldehyde (p-HPEA-EDA), and oleoside 11-methyl ester. Total tyrosol intake was calculated as the sum of all individual compounds; total daily energy intake was estimated using the EPIC standardized nutrient database [24]. The acute and habitual intakes of six foods/food groups (i.e., olives, olive oil, wine (including its red and white subtypes), and beer) with tyrosols content in Phenol-Explorer were used to calculate their correlations with urinary Tyr and OHTyr. Notably, data on the habitual intake of olives, and red and white wine were not available in all EPIC centers due to the differences in the specific DQs used in each center [17]. For example, data on olive intakes were only collected in Paris and Ragusa; red wine in Florence, Varese, Ragusa, and Turin; and white wine in Florence, Varese, Turin, Heidelberg, and Potsdam.

Other lifestyle information such as smoking status and physical activity was collected via standardized questionnaires at recruitment [18]. Data regarding age, body weight, and height were self-reported by study participants during the 24-HDR interview.

### 2.4. Statistical Analyses

The urinary excretions of Tyr and OHTyr that fell below the LOQ were replaced by half of the LOQ of the corresponding compound. Descriptive analyses of urinary tyrosols and acute and habitual intake of dietary tyrosols and foods included the number of samples < LOQ or non-consumers (as appropriate), as well as the median and 10th and 90th percentiles. A Kruskal–Wallis test was used to determine the differences in urinary and dietary tyrosols between different categories of demographic and lifestyle characteristics. A Spearman’s rank correlation analysis was used to assess the relationship between urinary tyrosol concentrations and dietary variables, including the acute and habitual intake of tyrosols and tyrosol food sources. For the habitual food intake, correlations with urinary tyrosols were only performed with foods having available records in all EPIC centers (i.e., olive oil, wine, and beer). To assess the strength of these correlations by controlling for potential confounders (i.e., BMI, age at recruitment, sex, center, smoking status, and total energy intake), a partial Spearman’s correlation analysis was performed. Differences between the acute and habitual intake of tyrosols and differences in the urinary excretion of Tyr and OHTyr among categories of lifestyle and sociodemographic variables were tested using a Mann–Whitney or a Kruskal–Wallis test, as appropriate. Similar analyses stratified by country, including descriptive and partial correlations analysis, were also performed. All results with *p* value ≤ 0.05 were considered statistically significant.

Statistical analyses were performed with the combination of SPSS (version 25.0; IBM, Armonk, NY, USA), R (version 4.2.1), and RStudio (2022.07.1+554).

## 3. Results

### 3.1. Urinary Excretions of Tyrosols

Table 1 shows the distribution of the 24 h urinary excretions of Tyr and OHTyr, and their sum (Tyr + OHTyr), according to the participants’ sociodemographic and lifestyle characteristics. Of the 419 individuals included in this study, only 18 (4.0%) and 1 (<1.0%) had urinary excretions of Tyr and OHTyr below the LOQ, respectively. The median urinary excretion of OHTyr (2.50 µmol/24 h) was higher than for Tyr (0.78 µmol/24 h). By country, the highest median urinary excretion of Tyr + OHTyr was found in Italy (5.81 µmol/24 h) followed by Germany (2.57 µmol/24 h) and France (2.47 µmol/24 h) (Table 1). The urinary excretion of Tyr and Tyr + OHTyr was higher in men, former smokers, and participants in the highest tertile of total energy intake (Table 1).

### 3.2. Intake of Tyrosols and Tyrosol-Rich Foods

The median acute intakes of Tyr, OHTyr, and total tyrosols (2.96, 0.67, and 12.5 mg/d, respectively) were higher than habitual intakes (1.94, 0.39, and 8.36 mg/d, respectively) (Table 2). The median acute intakes of 3,4-DHPEA-EDA and ligstroside were higher than habitual intakes, whereas the opposite occurred with 3,4-DHPEA-AC, 3,4-DHPEA-EA, p-HPEA-EA, p-HPEA-EDA, ligstroside aglycone, and oleuropein aglycone. However, as expected, the proportion (%) of consumers of tyrosol food sources was higher in habitual than in acute periods, such as for wine (acute: 44% and habitual: 88%), olive oil (acute: 45% and habitual: 75%), beer (acute: 14% and habitual: 67%), and olives (acute: 7% and habitual: 62%) (Table 2). The distribution of the acute and habitual intake of both dietary tyrosols and their food sources by country are shown in Supplementary Tables S1 and S2, respectively. Italy had the highest median acute and habitual intakes of total tyrosols with 30.4 and 15.9 mg/d, respectively, followed by France (11.3 and 8.74 mg/d, respectively), and Germany (2.95 and 2.71 mg/d, respectively). The acute and habitual intakes of olive oil and wine were higher in Italy, while the acute and habitual intakes of beer were higher in Germany.

### 3.3. Correlations between Urinary Excretions and Dietary Intake of Tyrosols

**Figure 1:**
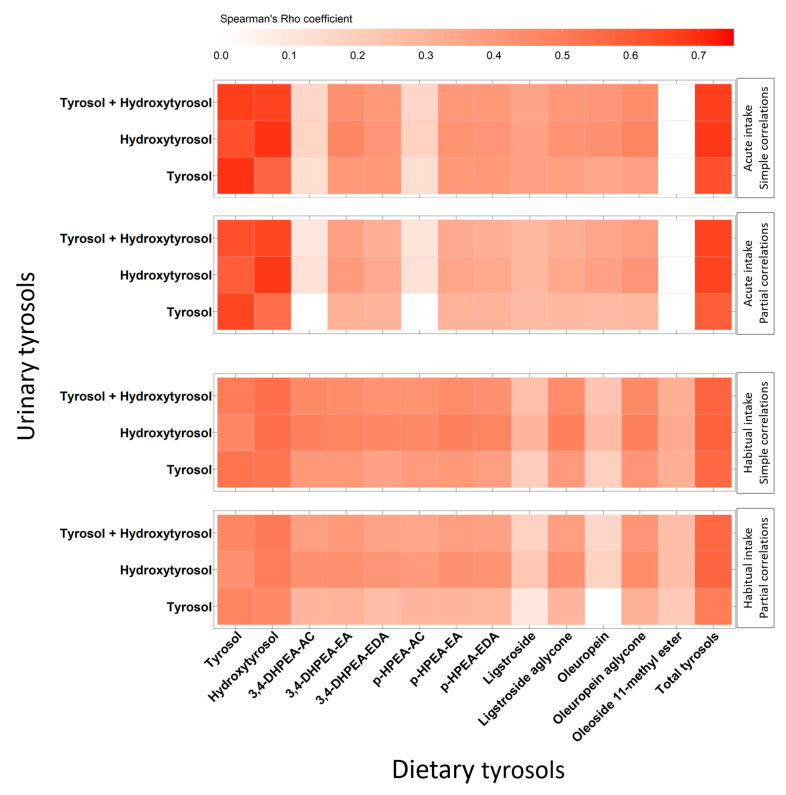
Heatmaps showing simple and partial Spearman’s correlations between urinary excretion and dietary intake (acute and habitual) of tyrosols. Abbreviations: 3,4-DHPEA-AC, hydroxytyrosol acetate; 3,4-DHPEA-EA, oleuropein-aglycone mono-aldehyde; 3,4-DHPEA-EDA, oleuropein-aglycone di-aldehyde; p-HPEA-AC, tyrosol acetate; p-HPEA-EA, ligstroside-aglycone mono-aldehyde; p-HPEA-EDA, ligstroside-aglycone di-aldehyde.

Significant simple and partial Spearman’s correlations between urinary and dietary tyrosols in all countries are illustrated in Figure 1. After controlling for sociodemographic and lifestyle factors, the partial correlations between the urinary excretions of Tyr, OHTyr, and Tyr + OHTyr, and the acute intakes of Tyr, OHTyr, and total tyrosols were relatively similar to those observed in simple correlations (rho_partial_ = 0.53–0.68). The partial correlations between the urinary excretions of Tyr, OHTyr, and Tyr + OHTyr, and the habitual intakes of Tyr, OHTyr, and total tyrosols were slightly lower (rho_partial_ = 0.43–0.57) than those observed in simple correlations. All individual dietary tyrosol derivates were weakly to moderately correlated with the individual and combined urinary excretions of Tyr and OHTyr, showing rho_partial_ correlation coefficients between 0.10 and 0.41 for acute intake, and from 0.10 to 0.44 for habitual intake (Figure 1). Full data on Spearman’s correlations, including coefficients and statistical significance between urinary and dietary tyrosols according to their acute and habitual intakes, are shown in Supplementary Tables S3 and S4, respectively. In terms of country, the partial correlation coefficients between the urinary excretions of Tyr + OHTyr and the acute and habitual intake of total tyrosols were as follows: France: 0.58 and 0.44; Italy: 0.57 and 0.41; and Germany: 0.62 and 0.56, respectively (Supplementary Tables S5 and S6).

### 3.4. Correlations between Urinary Tyrosol Excretions and Food Intakes

**Figure 2:**
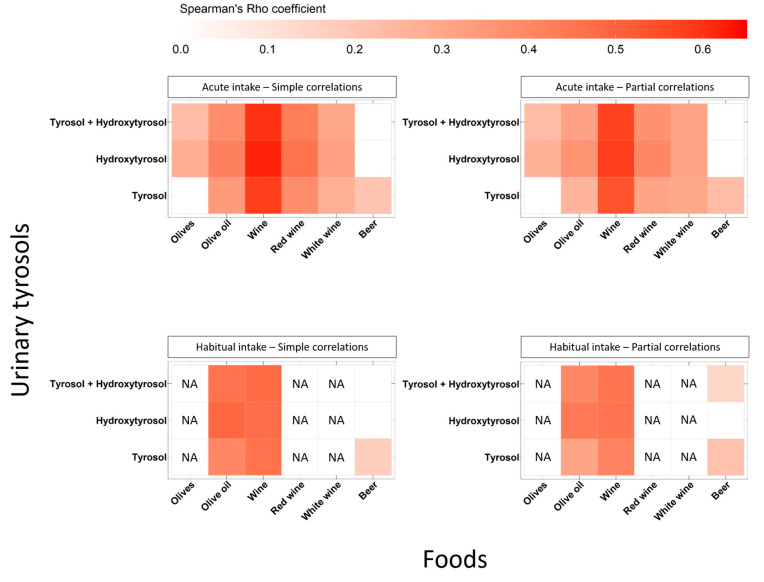
Heatmaps showing simple and partial Spearman’s correlations between urinary excretion of tyrosols and intake (acute and habitual) of selected tyrosol food sources. Abbreviations: NA, not available (i.e., correlation coefficients were not estimated due to lack of data on the habitual intake of these foods in all EPIC centers).

The urinary excretions of tyrosols were positively correlated with both the acute and habitual consumption of olive oil and wine, and the habitual intake of beer (Figure 2 and Supplementary Tables S3 and S4). The food group intake that most strongly correlated with the urinary excretions of tyrosols was wine (rho_partial_ = 0.54–0.58 for acute intake and rho_partial_ = 0.41–0.45 for habitual intake), followed by olive oil (rho_partial_ = 0.25–0.36 for acute intake and rho_partial_ = 0.31–0.44 for habitual intake) and beer (rho_partial_ = 0.23 for acute intake and rho_partial_ = 0.14–0.21 for habitual intake). The acute intake of beer correlated with the urinary excretions of Tyr (rho_partial_ = 0.23), while the habitual intake of beer correlated with urinary Tyr (rho_partial_ = 0.21) and Tyr + OHTyr (rho_partial_ = 0.14). The strongest correlations for each food intake with the urinary excretions of Tyr + OHTyr by country were as follows: the acute intake of olives and olive oil in Italy (both rho_partial_ = 0.21), whereas the habitual intake of olive oil only in Germany (rho_partial_ = 0.25); the acute intake of wine in all countries (rho_partial_~0.50), whereas its habitual intake in both Italy and Germany (both rho_partial_ = 0.44); and both the acute and habitual intake of beer in Germany (rho_partial_~0.30) (Supplementary Tables S5 and S6).

## 4. Discussion

In the current observational study, we found that the 24 h urinary excretions of Tyr, OHTyr, and their sum (Tyr + OHTyr) strongly correlated with their corresponding acute and habitual intakes. In addition, the urinary excretions of Tyr, OHTyr, and Tyr + OHTyr were weakly to moderately correlated with the acute and habitual intake of other individual tyrosol derivates, as well as with the acute and habitual intake of their principal food sources. All comparable correlations between urinary and dietary Tyr and OHTyr were stronger for acute than habitual intake. Furthermore, small differences in the correlations between urinary tyrosols and food sources were observed when correlation analysis was conducted by country.

To date, there is limited epidemiological evidence evaluating the use of the urinary excretions of Tyr and OHTyr as the potential nutritional biomarkers of tyrosol intake. Pooling data from several interventional studies in a systematic review, Pérez-Jiménez et al. determined that the 24 h urinary excretions of Tyr and OHTyr could be good biomarkers of their controlled intakes from olives, olive oil, and olive oil extracts, showing Pearson correlation coefficients r_Pearson_ = 0.72 for Tyr, and r_Pearson_ = 0.85 for OHTyr [16]. More recently, Aresta et al. found that the correlations between controlled amounts of Tyr and OHTyr ingested daily from extra virgin olive oil and those determined in 24 h urine were excellent (r > 0.90) [13]. Differences in the study design (i.e., observational vs. interventional), the distribution of tyrosol intake between populations, the methods used to estimate intake and the urinary excretions of Tyr and OHTyr, the food sources used for estimating dietary intake, and the correlation analysis method could explain the relatively lower correlation coefficients found in our study. Nevertheless, it is worth bearing in mind that, in our study, by using observational rather than experimental data, we found that the correlations between the 24 h urine excretions of Tyr and OHTyr and their intake ranged from moderate to strong (rho_partial_ = 0.53–0.68 and rho_partial_ = 0.43–0.50 for acute and habitual intakes, respectively). Thus, our results suggest that, within the setting of a free-living population, the 24 h urine excretions of Tyr and OHTyr could be used as biomarkers of the intake of these compounds, especially within an acute period.

In the present study, the excretions of Tyr, OHTyr, and Tyr + OHTyr were weakly to moderately correlated with the acute and habitual intake of 3,4-DHPEA-AC, 3,4-DHPEA-EA, 3,4-DHPEA-EDA, p-HPEA-AC, p-HPEA-EA, p-HPEA-EDA, ligstroside, ligstroside aglycone, oleoside 11-methyl ester, oleuropein, and oleuropein aglycone. Although these correlations could be expected due to common food sources, the magnitude of these might be influenced by tyrosol metabolism. After ingestion, the secoiridoid glycosides (i.e., oleuropein and ligstroside) and their aglycone derivates (i.e., oleacein (3,4-DHPEA-EDA) and oleocanthal (p-HPEA-EDA)) are subjected to an extensive first-pass hepatic metabolism in which, after a series of hydrolysis reactions, free forms of Tyr and OHTyr are released to systemic biological fluids [5,13]. As a result, only very low amounts of both secoiridoid glycosides and aglycones are detected in urine after their dietary intake [25]. We also calculated the sum of dietary Tyr and OHTyr (i.e., dietary Tyr + OHTyr) in order to compare the correlations between this combination and urinary tyrosols with those observed with total dietary tyrosols. As a result, we found that both acute and habitual intakes of Tyr + OHTyr and total tyrosols were similarly correlated with the individual and combined urinary excretion of Tyr and OHTyr. This suggests that the analysis of Tyr and OHTyr in urine alone would be sufficient to estimate the total intake and excretion of tyrosols.

As expected, the intakes of olives, olive oil, wine, and beer correlated with the urinary excretion of tyrosols. In fact, these correlations were consistent with previous studies demonstrating the presence of Tyr and/or OHTyr in human or animal urine and plasma after the consumption of the aforementioned foods. Table olives and olive oil are rich sources of Tyr and OHTyr; curiously, whereas in table olives, the amounts of OHTyr are usually higher than Tyr, in olive oil, the opposite occurs [26,27]. Likewise, regardless of the food source, it was observed that OHTyr excretions were usually higher than those of Tyr in urine and plasma [13,28]. In our study, these differences in tyrosol content in foods and their presence in biological fluids would partially explain why only urinary OHTyr (and then Tyr + OHTyr) correlated with the acute intake of olives, and why the correlations between urinary Tyr and olive oil intakes (acute and habitual) were weaker than those observed for urinary OHTyr (Figure 2). The intake of wine was the food source that most strongly correlated with the individual and combined urinary excretion of Tyr and OHTyr. Particularly, the correlations were stronger between urinary OHTyr and red wine, even when considering that OHTyr is frequently present in wines at lower excretions than Tyr [29]. However, the results obtained from controlled trials showed that alcohol from red wine could promote de novo OHTyr generation in vivo in humans by both improving Tyr bioavailability and its biotransformation into OHTyr via dopamine and tyramine oxidative metabolism [9,30]. Therefore, our finding could be partly explained not just by the higher wine intakes (both acute and habitual) among the participants, but also by a higher Tyr bioavailability and the subsequent potentially higher endogenous generation of OHTyr. Interestingly, it is possible that an analysis of Tyr and OHTyr conjugates in urine improves the correlations observed with the different food sources. It was previously shown that the controlled intake of olive oil or red wine produced a higher urinary excretion of glucuronide or sulfate conjugates, respectively [31]. The acute and habitual intakes of beer correlated with the urinary excretions of Tyr, and of Tyr and Tyr + OHTyr, respectively. These results were as expected since beer was less frequently consumed than wine and olive oil. Furthermore, the excretions of Tyr in beer were not negligible; nevertheless, they were much lower than those found in olives, olive oil, and wine [3].

Interestingly, after having stratified by country, we found that the correlations between the urinary excretions of Tyr + OHTyr and the intake of total tyrosols were more similar between countries using the 24-HDR (rho_partial_ = 0.57–0.62) than using the DQ (rho_partial_ = 0.41–0.56). Furthermore, the urinary excretions of Tyr + OHTyr were differently correlated with the acute and habitual intake of olive oil, wine, and beer between countries. The presence and strength of these last correlations are particularly consistent with those reported for the acute intake of foods in each country. Two assumptions may arise from these results. First, regardless of the geographical location and consequently of the dietary habits, the use of 24 h urinary excretions of Tyr + OHTyr would be more suitable to assess the acute rather than habitual intakes of total dietary tyrosols. Second, the use of 24 h urinary excretions of tyrosols as a biomarker of the consumption of olives, olive oil, wine, and beer is less specific than for total dietary tyrosol intakes, especially due to the variation in food sources by country. Moreover, it should be noted that because of the use of different DQs among EPIC centers, the information regarding the habitual intake of some tyrosol food sources was not available in all centers, especially for olives and wine subtypes.

The results of the current research may have clinical–nutritional implications. On the one hand, tyrosol measurements in systemic fluids partially reflect the exposure of individual or total tyrosols and can eventually replace or complement traditional dietary assessment as a means of reducing self-reporting inaccuracies and improving the reliability and objectivity of exposure measurements. Notably, the applicability of dietary (poly)phenol biomarkers in epidemiological studies relies on their ability to reflect the dose ingested, reliability over time, and the availability of appropriate analytical methods for their estimation in biospecimens [32]. Therefore, with this study, we added evidence to the potential use of urinary concentrations of Tyr and OHTyr as nutritional biomarkers of total tyrosols in large observational studies.

The main limitations of our study are related to the use of both self-reported questionnaires and the Phenol-Explorer database. In general, although self-reported dietary questionnaires are still a practical and affordable way to record individuals’ habitual diet, they may also introduce bias into the tyrosol intake assessment as a result of random and systemic measurement errors and may also restrict the variety of food items available in every study. However, it is worth mentioning that the dietary questionnaires used in this study were standardized or validated for the EPIC population. In addition, the lack of complete data on the habitual intake of specific tyrosol food sources may not only hamper the comparisons of correlations for their acute and habitual intakes of these foods with the urinary excretion of tyrosols but also may influence the estimation of the habitual intake of dietary tyrosols and their subsequent correlations with urinary tyrosols. For its part, Phenol-Explorer does not cover the variability of food tyrosol content and their bioavailability [33], which could contribute to an underestimation of tyrosol intake. Our study also had strengths, such as the availability of acute and habitual tyrosol intake, an adequate sample size, the 24 h urine sample, and accurate methodology to evaluate the urinary excretions of tyrosols.

## 5. Conclusions

Our study provided evidence that the 24 h urine concentrations of Tyr, OHTyr, and their sum can be proposed to efficiently assess total tyrosol intake, especially acute intake. The urinary excretions of Tyr, OHTyr, and their sum weakly correlated with dietary secoiridoid glycosides and aglycones, making them, therefore, ineffectual as nutritional biomarkers for these particular tyrosol precursors. Furthermore, urinary Tyr and OHTyr also correlated with the intake of wine and olive oil, and to a lesser extent, with beer; however, they could be considered as moderate or less specific biomarkers of these foods.

## Figures and Tables

**Table 1:** Distribution of 24 h urinary excretions (µmol/24 h) of tyrosol and hydroxytyrosol and their sum, according to sociodemographic and lifestyle characteristics of participants (n = 419) of the EPIC study.

		Urinary Tyrosol			Urinary Hydroxytyrosol			Urinary Tyrosol + Hydroxytyrosol		
Characteristics	N ^a^	n ^b^	Median (P10, P90)	*p* ^c^	n ^b^	Median (P10, P90)	*p* ^c^	n ^b^	Median (P10, P90)	*p* ^c^
All	419	18	0.78 (0.10, 5.33)		1	2.50 (0.75, 11.9)		0	3.51 (0.92, 16.7)	
Center				<0.01			<0.01			<0.01
Paris (France)	67	1	0.66 (0.12, 2.70)		0	1.60 (0.72, 6.56)		0	2.47 (0.93, 8.62)	
Florence (Italy)	45	0	1.60 (0.58, 5.84)		0	3.76 (1.47, 11.3)		0	5.49 (2.41, 16.7)	
Varese (Italy)	51	2	1.78 (0.24, 5.40)		0	3.78 (0.83, 9.76)		0	5.98 (1.08, 15.3)	
Ragusa (Italy)	17	0	2.56 (0.33, 7.11)		0	6.23 (2.72, 30.2)		0	12.4 (3.52, 36.2)	
Turin (Italy)	42	3	1.42 (0.35, 5.19)		1	4.52 (1.50, 20.4)		0	5.36 (1.94, 20.9)	
Naples (Italy)	20	1	0.67 (0.32, 1.91)		0	4.27 (1.35, 35.3)		0	5.58 (1.73, 36.7)	
Heidelberg (Germany)	59	5	0.52 (0.09, 6.10)		0	2.56 (0.77, 15.6)		0	3.09 (0.92, 19.8)	
Potsdam (Germany)	118	6	0.35 (0.07, 4.37)		0	1.40 (0.51, 4.18)		0	2.05 (0.69, 7.60)	
Country										<0.001
France	67	1	0.66 (0.12, 2.70)		0	1.60 (0.72, 6.56)		0	2.47 (0.93, 8.62)	
Italy	175	6	1.52 (0.32, 5.67)		1	4.08 (1.36, 15.6)		1	5.81 (1.80, 20.8)	
Germany	177	11	0.41 (0.08, 5.57)		0	1.69 (0.55, 7.47)		0	2.57 (0.70, 13.0)	
Sex				<0.01			0.12			0.01
Men	171	13	1.51 (0.10, 7.04)		1	2.84 (0.67, 13.0)		0	4.45 (1.00, 19.8)	
Women	248	5	0.65 (0.10, 3.13)		0	2.39 (0.77, 10.3)		0	3.12 (0.92, 13.2)	
Age (years)				0.12			0.30			0.38
<50	135	5	0.58 (0.08, 5.65)		1	2.27 (0.62, 15.7)		0	3.32 (0.74, 21.0)	
50–60	188	8	0.87 (0.14, 4.84)		0	2.84 (0.83, 10.8)		0	3.77 (1.12, 14.7)	
>60	96	5	0.75 (0.10, 5.50)		0	2.35 (0.76, 6.82)		0	3.37 (0.94, 12.3)	
BMI (kg/m^2^)				0.10			0.75			0.53
<25	201	10	0.81 (0.10, 5.38)		0	2.56 (0.76, 12.9)		0	3.41 (0.93, 17.8)	
25–<30	160	6	0.83 (0.13, 5.25)		1	2.57 (0.73, 10.5)		0	3.65 (1.08, 15.0)	
≥30	58	2	0.55 (0.08, 3.56)		0	2.19 (0.72, 9.46)		0	3.21 (0.86, 16.6)	
Smoking status				0.03			0.45			0.18
Never smoked	211	2	0.73 (0.09, 3.61)		0	2.56 (0.75, 10.8)		0	3.51 (0.92, 15.3)	
Former smoker	120	8	1.19 (0.14, 6.02)		0	2.51 (0.80, 11.9)		0	3.96 (1.07, 18.5)	
Current smoker	78	6	0.83 (0.09, 6.00)		0	2.07 (0.50, 14.0)		0	3.23 (0.74, 20.1)	
Acute total energy intake (kcal)				0.01			0.01			0.01
<1750	112	5	0.62 (0.10, 4.25)		0	2.09 (0.75, 7.49)		0	2.88 (0.88, 12.6)	
1750–2375	156	5	0.77 (0.09, 3.78)		0	2.63 (0.77, 11.4)		0	3.65 (0.93, 16.5)	
>2375	151	8	1.08 (0.13, 7.24)		1	2.95 (0.74, 12.9)		0	4.31 (1.08, 19.8)	

^a^ Number of participants in the corresponding category; ^b^ number of participants with urinary excretion of tyrosols < LOQ; ^c^ statistical significance from a Mann–Whitney or Kruskal–Wallis test, according to comparisons between two or more groups, respectively. Abbreviations: 24-HDR, 24 h dietary recall.

**Table 2:** Medians and 10th (P10) and 90th (P90) percentiles of acute and habitual intake of dietary tyrosols and their main food sources in participants (n = 419) of the EPIC study.

	Acute Intake		Habitual Intake		
	N ^a^/n ^b^	Median (P10, P90)	N ^a^/n ^b^	Median (P10, P90)	*p* ^c^
Dietary tyrosols, mg/d					
Tyrosol	419/34	2.96 (0.00, 27.9)	419/0	1.94 (0.17, 8.10)	<0.01
Hydroxytyrosol	419/64	0.67 (0.00, 8.41)	418/0	0.39 (0.03, 2.27)	<0.01
3,4-DHPEA-AC	419/272	0.00 (0.00, 0.34)	415/0	0.02 (0.00, 0.52)	<0.01
3,4-DHPEA-EA	419/113	0.40 (0.00, 4.06)	418/0	0.43 (0.04, 1.31)	<0.01
3,4-DHPEA-EDA	419/117	1.11 (0.00, 14.3)	418/0	1.04 (0.10, 3.30)	<0.01
p-HPEA-AC	4 19/272	0.00 (0.00, 0.00)	415/245	0.00 (0.00, 0.00)	<0.01
p-HPEA-EA	419/117	0.19 (0.00, 1.79)	418/0	0.20 (0.02, 1.03)	0.03
p-HPEA-EDA	419/117	0.66 (0.00, 7.69)	418/0	0.67 (0.06, 2.17)	<0.01
Ligstroside	419/130	0.03 (0.00, 0.47)	418/0	0.02 (0.01, 0.11)	<0.01
Ligstroside aglycone	419/117	0.13 (0.00, 1.29)	418/0	0.15 (0.01, 2.33)	<0.01
Oleoside 11-methyl ester	419/419	0.00 (0.00, 0.00)	417/203	0.00 (0.00, 0.01)	<0.01
Oleuropein	419/113	0.00 (0.00, 0.08)	418/0	0.00 (0.00, 0.68)	0.09
Oleuropein aglycone	419/113	0.32 (0.00, 3.96)	418/0	0.83 (0.03, 4.83)	<0.01
Total tyrosols	419/34	12.5 (0.02, 63.0)	419/0	8.36 (1.28, 24.2)	<0.01
Foods, g/d					
Olives ^d^	419/388	0.00 (0.00, 0.00)	84/32	0.40 (0.00, 4.68)	<0.01
Olive oil	419/232	0.00 (0.00, 27.9)	419/104	2.85 (0.00, 33.0)	<0.01
Wine	419/234	0.00 (0.00, 374)	419/51	55.3 (0.00, 324)	<0.01
Red wine ^e^	419/303	0.00 (0.00, 267)	155/27	62.5 (0.00, 333)	<0.01
White wine ^f^	419/346	0.00 (0.00, 138)	332/78	3.29 (0.00, 55.5)	<0.01
Beer	419/360	0.00 (0.00, 251)	419/138	5.52 (0.00, 260)	<0.01

^a^ Number of participants with available data of the intake for the corresponding compound or food; ^b^ number of participants reporting a null intake of the corresponding compound or food (i.e., non-consumers); ^c^ statistical significance from a Mann–Whitney test; ^d^ data on habitual intake of olives was only reported in Paris (France) and Ragusa (Italy) centers; ^e^ data on habitual intake of red wine was only reported in Florence (Italy), Varese (Italy), Ragusa (Italy), and Turin (Italy); ^f^ data on habitual intake of white wine was only reported in Florence (Italy), Varese (Italy), Turin (Italy), Heidelberg (Germany), and Potsdam (Germany). Abbreviations: 3,4-DHPEA-AC, hydroxytyrosol acetate; 3,4-DHPEA-EA, oleuropein-aglycone mono-aldehyde; 3,4-DHPEA-EDA, oleuropein-aglycone di-aldehyde; p-HPEA-AC, tyrosol acetate; p-HPEA-EA, ligstroside-aglycone mono-aldehyde; p-HPEA-EDA, ligstroside-aglycone di-aldehyde.

## Data Availability

The data presented in this study are available on request from the corresponding author.

## Supplementary Materials

The following supporting information can be downloaded at: https://www.mdpi.com/article/10.3390/antiox12030715/s1, Table S1: Distribution of acute dietary intake of tyrosols and food sources among three countries of the EPIC study; Table S2: Distribution of habitual dietary intake of tyrosols and food sources among three countries of the EPIC study; Table S3: Correlation coefficients for the relationship between 24 h urinary excretion and acute dietary intake of tyrosols and food sources in the EPIC study; Table S4: Correlation coefficients for the relationship between 24 h urinary excretion and habitual dietary intake of tyrosols and food sources in the EPIC study; Table S5: Partial correlation coefficients for the relationship between 24 h urinary excretion and acute dietary intake of tyrosols and selected food sources according to three countries of the EPIC study; Table S6: Partial correlation coefficients for the relationship between 24 h urinary excretion and habitual dietary intake of tyrosols and selected food sources according to three countries of the EPIC study.
